# An Open Forum for Computational Biology

**DOI:** 10.1371/journal.pcbi.0010005

**Published:** 2005-06-24

**Authors:** Michael Gribskov

It is my pleasure to welcome you to *PLoS Computational Biology*. The International Society for Computational Biology (ISCB) is proud to be, with the Public Library of Science, the co-sponsor of this journal. We believe that *PLoS Computational Biology* will rapidly become a leading journal in the area of computational biology and, as an official journal of ISCB, will be an important venue in which ISCB members will publish their findings and learn of the work of others, both ISCB members and nonmembers.

New journals appear at frequent intervals these days, and one may well ask: why a new computational biology journal? PLoS and ISCB feel that there is both need and interest in a journal with a new focus and new approach to computational biology. This feeling grows out of our recognition of the enormous changes that computation has wrought in both science and communication.

In contrast to the situation of only a decade or so ago, almost every area of biology is now affected and enhanced by computational studies. But until the appearance of *PLoS Computational Biology,* there has been no single publication with a focus on the important contributions of computational studies to the understanding of living systems. *PLoS Computational Biology* meets this need and provides a forum in which experimentalists and computational scientists can meet, exchange ideas, and develop new solutions to biological questions.

**Figure:**
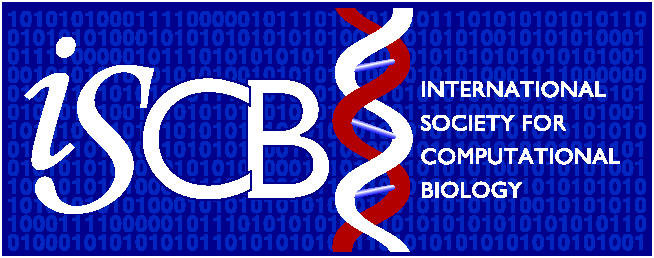


The revolution in communication that has grown out of the Internet and World Wide Web infrastructure provides the other motivating force behind the creation of a new journal. Free availability of protein and nucleic acid sequences, protein structures, and other biological data is critical to practitioners of computational biology—support of open-access journals is ISCB and PLoS launch an open-access journal with a new focus and new approach to computational biology. therefore a logical and natural progression for ISCB. Open access was a key factor in choosing PLoS as a partner. The ISCB believes that open access to scientific research is an important means of more rapidly disseminating scientific advances, and of communicating the findings of ISCB members to scientists around the world. Open access provides distinct advantages to scientific authors, including greater freedom to reuse and republish text, and greater availability of text for electronic data mining. In combination with rapid publication and high editorial standards, these provide compelling reasons for computational biologists to read and publish in *PLoS Computational Biology*.

In choosing *PLoS Computational Biology* as an official journal of the society, ISCB was also guided by the quality of PLoS, its board of directors, and senior editorial staff. It has been, and will continue to be, a pleasure collaborating with such an experienced and highly skilled group. PLoS brings great expertise in an experienced editorial staff and in developing the open-access publishing model. The experience and expertise of PLoS ensure that *PLoS Computational Biology* meets the highest production standards. Scientific leadership from ISCB, including the appointment of Philip E. Bourne, past-president of ISCB, as editor-in-chief will ensure the highest scientific standards. Moreover, the authors of each paper published in *PLoS Computational Biology* will be able to nominate one of their number to receive a complimentary membership to the ISCB. The Society and the journal thus work together to strengthen our community.

The participation of ISCB members will be critical to the success of *PLoS* *Computational Biology*. As authors, contributors, reviewers, and editorial board members, the members of ISCB will work with PLoS in building a successful journal. Together, ISCB and PLoS make ideal partners, and I am sure that you will join me in eagerly awaiting the first issues of *PLoS Computational Biology*.

